# Lentiviral standards to determine the sensitivity of assays that quantify lentiviral vector copy numbers and genomic insertion sites in cells

**DOI:** 10.1038/s41434-022-00315-8

**Published:** 2022-02-22

**Authors:** Guillaume Corre, Ababacar Seye, Sophie Frin, Maxime Ferrand, Kathrin Winkler, Cyril Luc, Fabien Dorange, Céline J. Rocca, Anne Galy

**Affiliations:** 1 Genethon, Evry, France; 2 Integrare Research Unit UMR_S951, Université Paris-Saclay, Univ Evry, Inserm, Genethon, Evry, France; 3 ART-TG, Inserm US35, Inserm, Corbeil-Essonnes, France

**Keywords:** Biotechnology, Gene therapy

## Abstract

With an increasing number of gene therapy clinical trials and drugs reaching the market, it becomes important to standardize the methods that evaluate the efficacy and safety of gene therapy. We herein report the generation of lentiviral standards which are stable, cloned human cells prepared from the diploid HCT116 cell line and which carry a known number of lentiviral vector copies in their genome. These clones can be used as reference cellular materials for the calibration or qualification of analytical methods that quantify vector copy numbers in cells (VCN) or lentiviral vector genomic integration sites (IS). Cellular standards were used to show the superior precision of digital droplet PCR (ddPCR) over quantitative PCR (qPCR) for VCN determination. This enabled us to develop a new sensitive and specific VCN ddPCR method specific for the integrated provirus and not recognizing the transfer plasmid. The cellular standards, were also useful to assess the sensitivity and limits of a ligation-mediated PCR (LM-PCR) method to measure IS showing that at least 1% abundance of a single IS can be detected in a polyclonal population but that not all IS can be amplified with similar efficiency. Thus, lentiviral standards should be systematically used in all assays that assess lentiviral gene therapy efficacy and safety.

## Introduction

Lentiviral vectors (LV) are widely used gene transfer vectors in gene therapy as they stably integrate and provide long-lasting expression of the therapeutic cassette in highly dividing transduced cells. LVs are used mostly for ex vivo gene therapy approaches with gene-modified hematopoietic stem/progenitor cells to treat various genetic disorders [1], or with gene-modified T cells such as CAR-T cells for cancer immunotherapy [2]. LV are also tested as injectable gene therapy products in neurological and ophtalmology indications [3]. Today, more than a hundred gene therapy trials using LVs are ongoing and several LV-based gene therapy drugs (Zynteglo, Kymriah, Abecma) have received marketing approval.

In spite of their advancement to clinical and market stages, there is still little standardization of the assays to measure lentiviral gene therapy efficacy and safety. A direct and broadly-applicable measure of lentiviral vector treatment potency is the level of transduction obtained in target cells as determined by an average number of integrated vector copies per cell (VCN). Longitudinal measures of VCN levels in blood cells are used to monitor the engraftment and persistence of gene-modified cells in patients treated by lentiviral gene therapy [4–6]. Coupled to transgene expression, VCN are indispensable values to assess the persistence or eventual silencing of transgene expression over time thereby providing a dynamic understanding of gene therapy effects. As observed in clinical trials, blood VCN levels can be relatively low (<0.1) in patients treated with transduced HSPCs but may increase over time to demonstrate a biological effect of the vector, as shown in Fanconi anemia A patients [6]. A precise and reproducible quantification of VCN can be challenging when small amounts of materials are used for instance in pediatric studies, or when small numbers of cells are analyzed such as sorted subpopulations of leukocytes or colony-forming unit cells (CFU-C). Thus, accurate and sensitive VCN assays are required with proper controls for quality assurance. Besides efficacy, the safety evaluation of gene therapy with LV is also not well standardized. One aspect of safety evaluation relies on the analysis of the LV genomic insertion sites (IS) in target cells. The pattern of genomic IS of HIV-1-derived LV is well-characterized [7] and its mapping can serve not only to confirm the identity of the vector and to characterize the transduced cells, but also to verify the absence of specific insertional configurations that can lead to insertional toxicity [8]. Based on previous reports of insertional mutagenesis with retroviral vectors [1] a threshold of 20% single clone abundance is usually proposed as a safety threshold below which a single clone is not considered to dominate [4, 5] but the sensitivity of vector IS methods is not well known. Thus, cellular reference materials with known VCN and known IS would be useful to assess and to improve the methods that evaluate gene therapy safety and potency.

Previous efforts have been made to generate such lentiviral reference materials. Lentiviral standards are homogenous cell lines with stable numbers of VCN and IS which have been established by several laboratories, including our own, to calibrate their VCN or LV titration assays and which have been in some cases proposed as international standards [9–12]. Our laboratory has previously cloned HT1080 cells transduced with a green fluorescent protein (GFP)-expressing lentiviral vector and containing 1, 3, or 8 copies of integrated vector [9]. Such clones demonstrated a high correlation between VCN and transgene expression measured by GFP mean fluorescence intensity in flow cytometry, as reported by others [10]. Such clones served in preclinical studies to assess the sensitivity of several DNA extraction and PCR methods to detect VCN in individual CFU-C and to validate VCN results in clinical gene therapy trials [4, 6, 13]. However, over time, we observed a drift in the VCN values obtained by quantitative PCR (qPCR) in HT1080 cell-derived lentiviral standard cell lines. In accordance with a reported frequent pseudodiploidy and instability in these cells (as per ATCC), we found abnormal karyotype in batches of these HT1080 derived cell clones, probably caused by extensive cell culture and variant selection [14]. These data prompted us to generate new lentiviral standards using a diploid cell line HCT116. Clones of transduced HCT116 cells with 1, 2, and 3 VCN were selected and used to determine the performance of several PCR methods used for VCN. Results demonstrate the superior performance of ddPCR over qPCR and helped to develop an improved VCN assay using a ddPCR method that specifically measures provirus integration and not plasmid contaminants. In addition, these new lentiviral standards were used to assess the sensitivity of ligation-mediated PCR (LM-PCR)-based method to determine vector IS and its limits as not all IS could be determined with equal efficiency.

## Materials and methods

### Cells

HCT116 colorectal human cells (American Type Culture Collection—ATCC, Manassas, VA) which are near diploid with 45 median chromosome count were cultured at 37 °C and 5% CO_2_ in complete DMEM medium (DMEM with 4.5 g/L glucose (Gibco, Waltham, MA) supplemented with 10% (v/v) fetal calf serum (Eurobio, Les Ullis, France), 1% (vol./vol.) GlutaMAX^®^ (Gibco) and 1% (v/v) penicillin/streptomycin (Gibco)). A subculture of HEK293T human embryonic kidney cells was derived at Genethon and used to produce LV for research or for clinical use as previously reported [4]. Such cells were derived from a working cell bank which was authenticated and tested negative for mycoplasma.

### Lentiviral vector production and titration

A VSV-G pseudotyped self-inactivating lentiviral vector coding for the truncated human nerve growth factor receptor (dNGFR/CD271) under the control of a human phosphoglycerate kinase (PGK) promoter was produced by transient transfection of 293T cells using calcium phosphate and 4 plasmids (pRRL-hPGK-dNGFR-WPRE, HIV-1 gagpol, HIV-Rev, and VSV-G plasmids). The harvested particles were concentrated about 500 fold by ultracentrifugation (50,000 × *g*, 2 h, 12 °C) suspended in phosphate buffered saline and cryopreserved at −80 °C. The infectious titer of the vector, determined on HCT116 cells using qPCR as previously reported [9], was 2.1E9 infectious genome (IG)/mL.

### Magnetic cell sorting and flow cytometry

Cells expressing the dNGFR transgene were positively selected by CD271 magnetic bead cell sorting (Miltenyi Biotec, Bergisch Gladbach Germany) using manufacturer’s instructions. Transgene expression was measured by flow cytometry using the LSR II cytometer (BD Biosciences Franklin Lakes, NJ), anti-CD271 antibodies (CD271 NGFR APC # 130-110-080, Miltenyi Biotec) and 7AAD (Sigma-Aldrich, St. Louis, MO) to exclude dead cells.

### DNA extraction and PCR methods

DNA was extracted using the Wizard Genomic DNA purification kit (Promega, Madison, WI USA) according to the manufacturer recommendations. Vector copy number (VCN) was measured by quantitative real time PCR (qPCR) or by digital droplet PCR (ddPCR). The names of the primers and probes are indicated in italics and the sequences are listed in Supplementary Table S1. Amplification of the human ALB gene with *Alb.fw*, *Alb.rv* and *Alb.pr* was used to determine the number of diploid genome. The amplification of the vector HIV-1 PSI sequence with *PSI.fw, PSI.rv*, and *PSI.pr* or LTR- sequence with *PRO.fw, PRO.rv*, *PRO.pr* were respectively used to determine the vector copies. For qPCR, the reaction was performed using 100 ng of gDNA per reaction using the Light Cycler 480 device (Roche Life Science Penzberg, Germany) according to the manufacturer recommendations (ABsolute qPCR ROX Mix, Thermo Scientific, Waltham, MA USA). A qPCR standard curve was used to convert Ct values to copy numbers by amplifying serial dilutions of a plasmid containing equimolar ratios of the PSI and ALB targeted sequences. For ddPCR, the reaction was performed on the Biorad system (Biorad QX200 autoDG and PCR systems Hercules, CA USA) according to the manufacturer recommendations using 3 units of the HaeIII restriction enzyme (New England Biolabs (NEB), Ipswich MA USA) in the mix (Biorad ddPCR Supermix for Probes (No dUTP) and 40 ng of gDNA per reaction unless indicated otherwise.

### Vector IS analysis

Vector IS were identified using a Linker-Mediated PCR (LM-PCR) technique with paired-end Illumina sequencing to amplify the junction between the integrated provirus 3′LTR and the genomic DNA at IS. The sequences of primers and oligonucleotides used are found in Supplementary Table S1. The gDNA (500 ng in 50 µl) was sonicated for 5 cycles of 15″ON/30″OFF (Diagenode, Bioruptor Pico, Liege, Belgium) to obtain a mean fragment size of 600 bp. Fragmented DNA was then end-repaired and a protruding 3′ A was added following the manufacturer recommendations (NEB). The linker was assembled by mixing oligos *Linker*+ and *Linker*− to a final concentration of 20 µM in 10 mM Tris, pH 7.5–8.0, 50 mM NaCl and 1 mM EDTA. The mix was heated at 95 °C for 5 min and slowly cooled down to room temperature at a rate of 1.5 °C/min and stored at −20 °C. The double stranded linker was ligated to the repaired fragmented DNA with a molar ratio of 5:1 (NEB) and the ligation product purified using spin columns using a 5:1 buffer dilution to eliminate fragments smaller than 200 bp (Macherey Nagel, Dueren Germany). A first PCR was performed for 30 cycles to amplify vector/genome junctions with primers *VISA1.vector* and *VISA1.linker* (TM = 64 °C) using 4 µl of DNA in 25 µl reaction volume (NEB). A *Blocking oligo* (10 µM) containing bridged nucleic acid (BNA) bases was used to suppress the generation of unwanted sequences elongated from the 5′LTR end of the provirus, thereby increasing the number of reads containing the vector/junction from the 3′LTR as described [15]. After purification, 2 µl of DNA was amplified by 20 cycles of nested PCR in order to add Illumina NEXTERA adaptors and 4 bp barcodes using primers *VISA2.TAGx.vector*, *VISA2.linker*, and 10 µM of the *blocking oligo*. After PCR, amplicons ranging from 400 to 1 kb were gel purified and sequenced using either Sanger sequencing (Genewiz, Leipzig Germany) after cloning of PCR products in Top10 E.coli (Invitrogen, Whaltham, MA) or MiSeq Illumina paired-end sequencing (301 bp, 2E6 reads, IGATech, Italy). For the PCR verification of the vector/genome junction, we used specific primers recognizing gDNA sequences around the identified IS and the *VISA1.vector* primer with the OneTaq DNA polymerase (NEB). Amplicon were size-selected on gel and sequenced by Sanger sequencing using the *VISA2.vector* primer (Genewiz, Germany).

### Data processing and statistics

To identify IS from sequencing data, raw reads from Sanger sequencing or from Illumina sequencing were first demultiplexed (cutadapt v2.2) based on the sample TAG added during the nested PCR and both the LTR and the linker sequences were trimmed (cutadapt v2.2). Resulting sequences longer than 20 bp and not containing the proviral or plasmid sequences were aligned on the reference genome (hg19, bowtie2 v2.3.4.2) and insertion point inferred from the alignment coordinates. Reads mapping to the same IS were aggregated and the number of different sonication fragments was estimated using the sonic-Length method [16]. Each IS was then annotated using the gencode database release 19. The availability of the code used for this study is subject to restrictions.

Data processing, statistics and figures were produced using the R software 3.6.0. Information about sample size and statistical tests are found in the figure legends.

## Results

### Generation of cellular clones serving as lentiviral standards

**Fig. 1:**
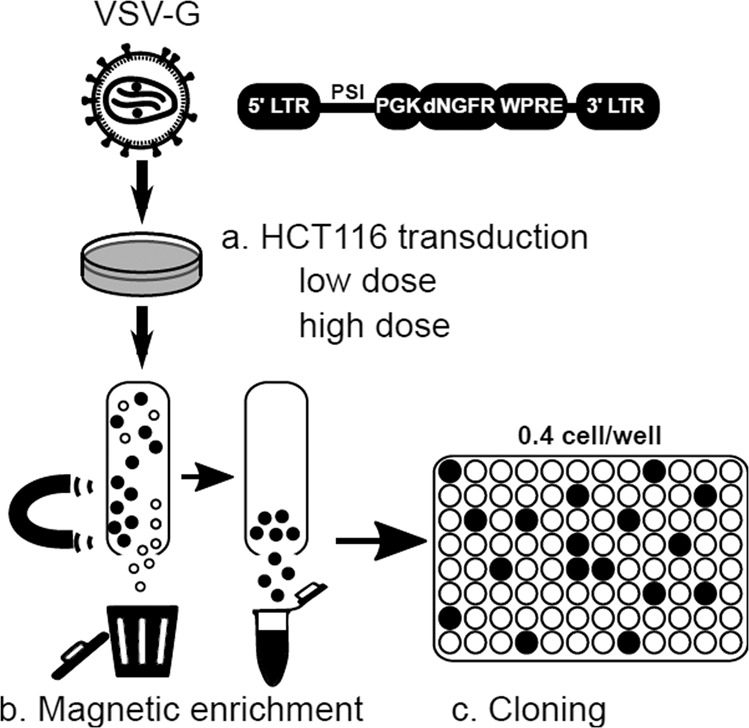
Generation of lentiviral standard cellular clones. HCT116 cells were transduced with various doses of an advanced-generation LV encoding the dNGFR transmembrane protein. The low vector dose corresponds to a multiplicity of infection (MOI) 0.05–0.5. The high doses corresponds to MOI 1–10. Cells expressing the transgene were enriched by magnetic cell sorting before cloning by limiting dilution. Following cell expansion, the clones were characterized and cryo-conserved.

**Fig. 2:**
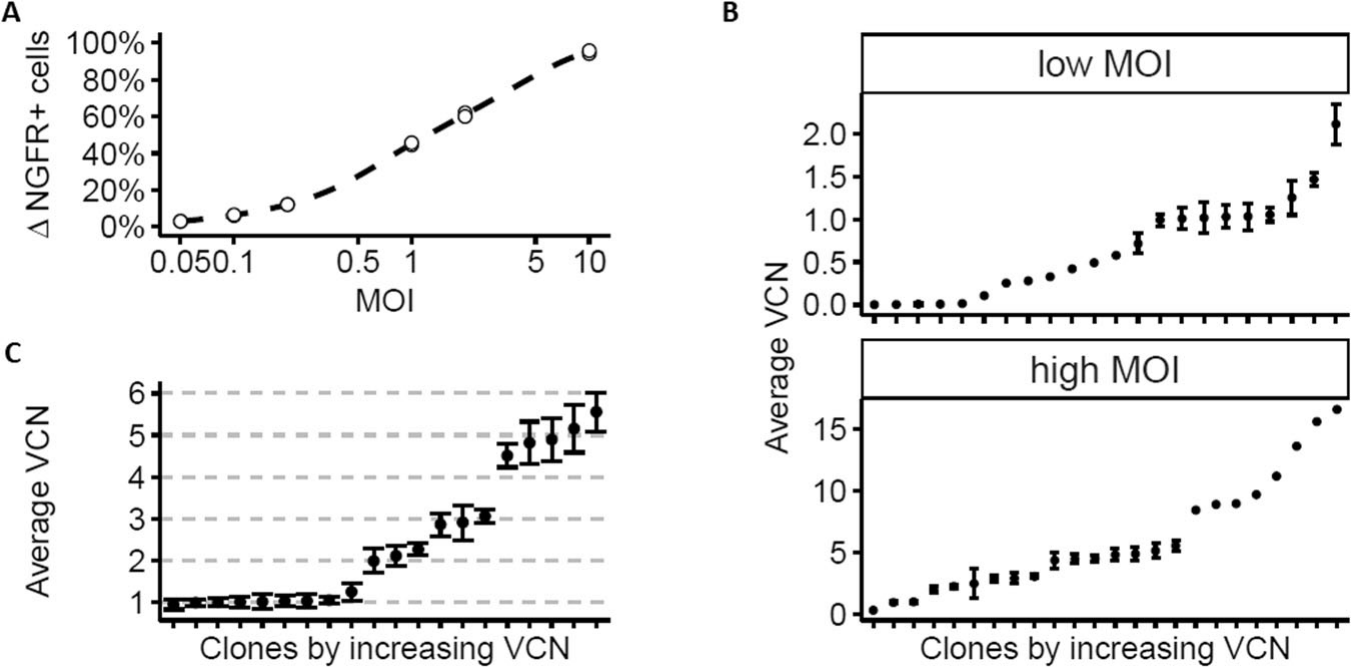
Cell transduction efficiency and clone screening. **A** Expression of the transgene was measured by flow cytometry 4 days after transduction performed in duplicate, showing that the proportion of transduced cells increases accordingly to the MOI. Dots show the result of each duplicated transduction. **B** Measure of VCN by qPCR PSI-ALB, in all the clones obtained from the pool of low vector dose (MOI 0.05–0.5) ranging up to VCN = 2.0 (top panel) or high vector dose (MOI 1–10) (ranging up to VCN = 17). Data represent means with error bars denoting mean 95% confidence intervals with 5 to 20 independent qPCR runs per clone. **C** Measure of VCN by qPCR PSI-ALB in a selection of clones with 1 to 6 VCN which were selected and further characterized. Data represent means with error bars denoting mean 95% confidence intervals with 5 to 20 independent qPCR runs per clone.

Cellular lentiviral standards were prepared from HCT116 cells that are known to be stably near diploid and have been used for more than 15 years in our laboratory for infectious titration assays of LV [17]. HCT116 cells were transduced with an advanced-generation pRRL lentiviral vector encoding the truncated nerve growth factor receptor (dNGFR) and schematized in Fig. 1. To obtain cells with a broad range of vector copies, different amounts of vector were used from 2.5E4 to 5E6 infectious genomes (IG)/mL which is equivalent to a multiplicity of infection (MOI) 0.05 to 10. These conditions generated a broad range of transgene-positive cells reaching almost 100% at the highest dose of vector used, as measured by flow cytometry 4 days after transduction (Fig. 2A). Two pools of cells were constituted, one transduced between MOI 0.05 and 0.5 and another between MOI 1 and 10, to obtain cells with low or high VCN. After an amplification period of 10 days dNGFR transgene-positive cells were enriched in each of the low and high MOI pools using immuno-magnetic selection (about 73% and 93% transgene-positive cells, respectively) and the positively-selected cells were cloned, yielding 22 and 24 clones respectively. Clones were amplified for three additional weeks before measuring their VCN using an already reported duplex qPCR technique amplifying the HIV-1 PSI vector sequence and the human ALB cellular gene [9] (Fig. 2B). As expected, the range of VCN of the clones was correlated to the concentration of vector used and to the purity of the starting population, ranging from 0 to 2.5 for the low MOI condition and 1 to 17 for the high MOI condition (Fig. 2B). Expression of the dNGFR transgene was positive but levels were not indicative of VCN levels due to the high membrane stability of this transgene (data not shown). A panel of clones with VCN values comprised between 1 and 5 was repeatedly tested for VCN, between 5 and 20 tests each, showing reproducible results (Fig. 2C). Within this panel, clones KS10, KS39, KS40 with respectively 1, 2, and 3 VCN were selected for further characterization as these VCN values usually correspond to those expected in ex vivo gene therapy trials. These three selected clones confirmed that they had comparable chromosome counts to the parental cell line at a median close to 45 as described for HCT116 cells (Supplementary Fig. S1).

### Use of cellular clones to assess the precision and the sensitivity of VCN quantification by qPCR or by ddPCR

**Fig. 3:**
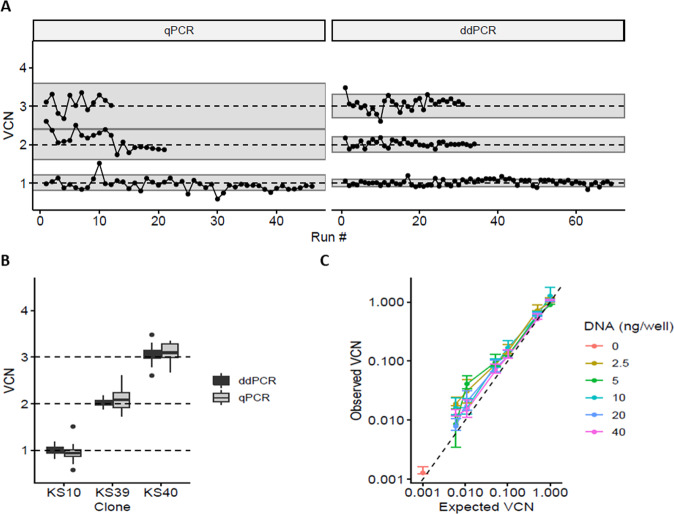
Comparison of VCN quantification methods on three selected reference clones. Clones KS10, KS39 and KS40 with 1, 2 or 3 VCN respectively, were extensively tested for VCN over time using either qPCR or ddPCR. **A** Longitudinal quantification of VCN with both methods over a period of 4 years indicated by the different tests (runs) and corresponding to more than 60 independent runs for clone KS10 (1 copy) and up to 9 gDNA batches tested. Batch-to-batch variation was limited (≤6%). Gray ribbons represent a target variation of 20% (qPCR) or 10% (ddPCR) around the expected VCN values. **B** The aggregation of all VCN obtained in A show that ddPCR is twice more accurate than qPCR with a coefficient of variation (CV) between 3 and 8%. **C** Clone KS10 with a single integrated provirus was used to challenge the ddPCR sensitivity using decreasing amount of gDNA (2.5 to 40 ng per well) and decreasing VCN by mixing with gDNA of untransduced cells down to 0.005 VCN. Each combination of DNA quantity/VCN was tested independently 4 times. Data represent mean ± standard error of the mean (sem). For a log/log representation of data, 0.001 was added to all values.

A ddPCR method was developed to improve the performance of VCN assays and to reduce the factors of variability of the method. The ddPCR quantifies the absolute amount of target DNA in the reaction and does not require a standard curve whereas the qPCR method quantifies VCN in relation to dilutions of a standard material comprising both the vector and cellular sequences. This standard curve needs to be prepared and validated regulary, thus introducing a variability factor. We developed a VCN assay by ddPCR using the same PSI and ALB primer sequences than qPCR and the performance of the assays was compared. The three selected lentiviral standards were used systematically in the qPCR and ddPCR experiments performed in the laboratory, representing up to 50 runs over a period of 4.5 years for the clone with 1 VCN (Fig. 3A). To detect eventual drifts, a longitudinal follow-up of the VCN results was charted with an alert range arbitrarily set at 20% of target value for qPCR and 10% for ddPCR as illustrated on Fig. 3A. Both qPCR and ddPCR methods were found to be accurate, giving the expected average VCN for each clone, but the ddPCR method was clearly more precise as illustrated by Fig. 3A, B and shown in Table 1. With the three standards, a coefficient of variation varied between 4–8% for ddPCR and 10–15% for qPCR. Lentiviral standards also served to demonstrate a satisfactory reproducibility of ddPCR on different gDNA batches: KS10 = 1.04 ± 0.06 (*n* = 9 batches), KS39 = 2.02 ± 0.08 (*n* = 5 batches), and KS40 = 3.1 ± 0.12 (*n* = 4 batches).

**Table 1:** Comparison of qPCR and ddPCR to measure VCN using the PSI primer set in clones KS10, KS39, and KS40 during longitudinal studies.

Clone	Technique	# tests	mean	sd	CV	SEM
KS10	qPCR	46	0.94	0.14	14.89	0.02
KS10	ddPCR	69	1.01	0.07	6.93	0.01
KS39	qPCR	21	2.11	0.24	11.37	0.05
KS39	ddPCR	34	2.02	0.08	3.96	0.01
KS40	qPCR	12	3.08	0.21	6.82	0.06
KS40	ddPCR	31	3.06	0.17	5.56	0.03

*sd* standard deviation, *CV* coefficient of variation, *SEM* standard error of the mean.

The single copy clone KS10 was used to estimate the limit of detection (LOD) and the limit of quantification (LOQ) of the ddPCR assay according to the amount of gDNA per reaction and according to the VCN value. Diluting the gDNA from clone KS10 in water provided a range of gDNA per reaction from 40 ng down to 2.5 ng gDNA. Diluting the gDNA of clone KS10 in gDNA from untransduced cells provided a range of VCN from 1 to 0.005 VCN. Figure 3C shows that the LOD of the ddPCR is 0.005 VCN. The LOQ was found to vary according to the amount of gDNA per reaction. When using 20–40 ng gDNA per reaction, the LOQ was estimated to be 0.01 VCN to obtain the same accuracy and precision than with 1 VCN. Below 0.01 VCN, the measure becomes impacted by low DNA amount probably due to the very low number of positive droplets and to other variability factors.

### Development of a more specific method for VCN determination

**Fig. 4:**
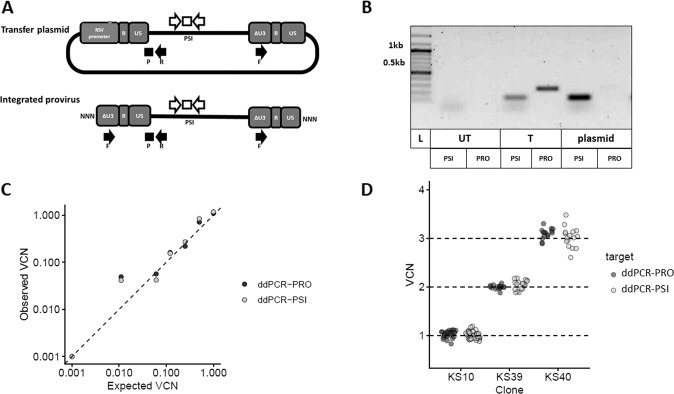
Development of a ddPCR specific for integrated provirus VCN. **A** Representation of sequence recognition by the provirus (PRO) (black arrows) or vector PSI (white arrows) primers and probes on the lentiviral transfer plasmid (top) or integrated provirus (bottom). **B** Agarose gel detection with 100 bp ladder (L) of the products of PCR amplification of DNA from plasmid, untransduced cells (UT), transduced cells (T) with PRO or PSI primer sets. **C** Correlation between expected and observed VCN using dilutions of gDNA from clone KS10 and ddPCR with PSI or PRO primer sets using 40 ng of DNA per reaction. Each dot represents the average of a duplicate PCR measure. **D** Individual ddPCR results with PSI and PRO primer sets on the three lentiviral standard clones. Each dot represents the average of a duplicate PCR measure.

A confounding factor in measures of VCN is to detect not only the sequences of vector integrated in the genome of transduced cells but also traces of the transfer plasmid that is used for vector production and which remains as a residual contaminant in most vector preparations. As schematized in Fig. 4A, the use of the PSI primers do not discriminate between provirus and plasmid as confirmed by a positive PCR result (Fig. 4B). For a more specific VCN measure, we designed and tested a set of primers amplifying the region between the U3-deleted LTR and the vector with a probe spanning the junction between the U5 region of the LTR and the vector (Fig. 4A) (Supplementary Table 1). Thus, as confirmed by results in Fig. 4B, PCR amplification is obtained when the LTR is duplicated during reverse transcription in transduced cells but not with the plasmid. The 3 lentiviral standards tested by ddPCR-PRO using the provirus-specific primers/probe set provided the expected VCN values as shown in Table 2. The sensitivity of the ddPCR-PRO seeemed to be comparable to that of ddPCR-PSI based on serial dilutions of the 1 VCN− clone and using an optimal amount of 40 ng gDNA per reaction (Fig. 4C). The accuracy and precision of the ddPCR-PRO was also at least as good as that of the ddPCR-PSI as shown in Fig. 4D.

**Table 2:** Statistics of VCN quantification with the different methods, and genomic IS coordinates of the lentiviral standards.

Clone	VCN ddPCR_PSI	VCN ddPCR_PRO	IS position
KS10	1.01 ± 0.07 (*n* = 42)	1.03 ± 0.07 (*n* = 27)	chr17_1719890_+
KS39	2.04 ± 0.09 (*n* = 18)	1.99 ± 0.04 (*n* = 16)	chr22_41230375_− chr4_188092607_−
KS40	3.02 ± 0.21 (*n* = 16)	3.09 ± 0.11 (*n* = 15)	chr2_74442451_+ chr5_179151158_+ chr8_144812321_+

VCN of each clones were measured using the indicated methods in non-paired runs. The mean VCN values ± sd are indicated with the number of tests performed, in parenthesis. The genomic coordinates of the unique IS identified are from the hg19 reference genome (chromosome_position_strand).

### Characterization of vector IS in selected cellular clones

To further characterize the lentiviral standards, the vector genomic IS was identified in each clone using LM-PCR followed by paired-end sequencing of fragments containing vector-genome junctions and annotation of the position. Results for each clone confirmed an expected number of IS based on the VCN values (Table 2). Genomic positions with multiple fragment sizes were considered as putative IS and validated using PCR with specific primers between the 3′LTR and the gDNA region identified by LM-PCR. All PCRs gave the expected amplicon size for the specific clone and the amplicon sequencing confirmed that the expected vector-genome junction was present. All but one IS were annotated in gene bodies, as expected from the reported insertion profile of lentiviruses [7].

### Use of cellular clones to assess the limit of detection of vector IS

**Fig. 5:**
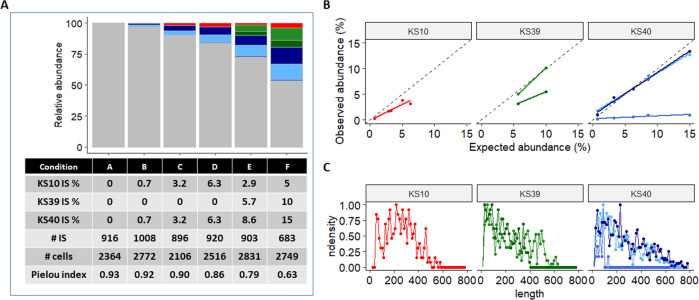
Estimation of the LM-PCR sensitivity limit. Different amounts of gDNA from the three reference clones were spiked with gDNA from a polyclonal population of transduced cells in order to simulate different levels of individual IS clonal abundances (see Supplementary Table S2) and vector IS analysis was performed. **A** Stacked bar graph representing the IS observed in each condition. The number of unique IS is indicated below. The unique IS that represent less than 2% of all unique IS are shown in gray, and IS from clone KS10, KS39, and KS40 are represented respectively in red, green and blue using different hue saturation for the different IS of each clone. The number of cells is calculated by sonic-length method [15] and the Pielou evenness index (Shannon index normalized to the number of IS analyzed) is indicated. **B** Representation of the recovered and expected abundance for each IS of the three lentiviral standards. The color scheme of (**A**) is used in the graph lines to represent each of the IS of the different clones. **C** Distribution of the fragment length distribution for each of the IS in the three lentiviral standards.

To estimate at which level of sensitivity the LM_PCR protocol can detect the dominance of a single IS, we simulated various levels of clonal abundance (from 0.7 to 15% of IS) by diluting and mixing the gDNA of the 3 lentiviral standard clones in various amounts (from 1 to 30% of total gDNA) with a bulk population of highly polyclonal transduced HCT116 cells cells containing an average VCN 1.5 (Supplementary Table S2). The analysis of an acceptable number of IS and of cells confirmed that the experimental design provided the expected variations in the relative abundance of IS from the different clones as shown in Fig. 5A, B. As the relative abundance of the spiked clones increased, the evenness of each population decreased as shown by decreasing values of the Pielou index, a normalized Shannon diversity index which correlates with a lower degree of polyclonality. The results show that the LM-PCR protocol is sensitive as it can detect an IS contributing between 0.7 and 3% of a total polyclonal population as shown in conditions B and C (Fig. 5A). The results also show that not all IS can be quantified in equally efficient manner. Indeed, the IS of clone KS10 is detected when it represents 3% of the population whereas those of clones KS40 are detected when they each represent 0.7% of IS. In addition, 1 out of the 3 IS of clone KS40 is not well detected. While a correlation was observed between the relative and expected abundance for some IS, there was a 2-fold under-estimation of 1 IS of clone K39 and a strikingly strong reduction in the detection of 1 IS of clone KS40 (Fig. 5B). This indicates that the relative abundance of specific genomic positions may be regularly underestimated by this method. As seen in Fig. 5C, the distribution of fragment length obtained after LM-PCR is generally broad and can reach up to 800 bp as expected from the fragment length distribution after sonication. However, the amplification profile of this particular IS of clone KS40 is biased toward small sizes (<200 bp) and a similar observation is made with 1 of the IS of clone KS39. In such cases we observed an AT rich region at the same distance from the IS indicating that nucleotide composition, particularly repeated sequences, may impair the polymerase processivity in those regions. In addition, specific amplification of this particular IS failed when using primers between the 3′LTR and the genomic DNA. Only amplification from the 5′LTR gave the right amplicon (size and sequence) which may confirm a particularly difficult to amplify DNA composition or structure in the downstream region of IS. Thus, lentiviral standards have proven useful to assess the sensitivity and as well as to understand the limits of this vector insertion site method.

## Discussion

We herein report the generation of new lentiviral standards and their use to calibrate, to assess and to improve some of the quality control assays for gene-modified cells.

The accurate and precise quantification of VCN in transduced cells is key to evaluate the efficacy of gene therapy with LV. Many clinical studies have used qPCR for measures of VCN [4–6, 13] but our results show that the ddPCR technique is more performant. The ddPCR technique provides a direct quantification of DNA without a standard curve and is therefore less susceptible to variation. Already, ddPCR has been applied to lentiviral vector titration showing a broad dynamic range for the quantification between 30 to 7700 copies of HIV sequence per reaction as measured on a plasmid [11]. The ddPCR technique was also used by others for VCN measures and reportedly provides similar results as qPCR [10]. In our case, we show that ddPCR with PSI-ALB sequences enables a VCN measure on human cell gDNA using 20–40 ng per reaction with a LOQ at 0.01 VCN and LOD at 0.005 VCN. Considering that a cell contains about 6 pg gDNA, our ddPCR method has similar performance to that reported for lentiviral titration [11] also confirms and extends findings by others [10] demonstrating that ddPCR has superior accuracy, precision and a high reproducibility rate for VCN determination compared to qPCR. Furthermore, the method could be improved by developing a new set of specific primers that only detect the integrated provirus and not plasmid sequences. Plasmid sequences are unwanted contaminants from the lentiviral production which can lead to an over-estimation of VCN in the target cells especially when it is necessary to test transduced cells very shortly after cell transduction or when using research-grade batches of lentiviral vector that are not extensively purified (data not shown). The new ddPCR-PRO enabled a more specific and seemingly equally sensitive and accurate measure of VCN than the previous ddPCR-PSI. With further validation, this new provirus-specific VCN assay could be used to monitor VCN in preclinical studies as well as in gene therapy patients blood cells.

An important evaluation of the safety of integrative vectors relies on the identification and relative quantification of genomic insertions in the genome of target cells to survey for clonal dominance. Indeed, since the first occurences of secondary effects due to retroviral vector integration near oncogenes in early gene therapy clinical trials [18] and the subsequent observations of clonal dominance in lentiviral gene therapy trials [8], a survey of vector genomic IS is recommended by guidelines for preclinical development of gene therapy products by agencies such as the European Medicines Agency. A commonly-accepted threshold of 20% can define clonal dominance by a specific IS compared to all other IS. The sensitivity of methods used to measure IS are not always specified but can be determined using a mixture of polyclonal cells with known amount of cells containing known vector copies number [15]. Using a similar approach, we simulated various levels of IS abundance and estimated that our LM-PCR method can identify a single insertion site representing down to 0.7% of the total abundance which makes it pertinent to assess clonal dominance in gene therapy. However, the use of the different lentiviral standards reveals a limitation of the LM-PCR IS analysis method which underestimates the relative abundance of particular IS. It has already been reported that LM-PCR IS analysis is not the most efficient method to estimate the number of integrations in transduced cells. Compared to a vector DNA barcoding sequencing method, LM-PCR underestimates the number of different integrants in monkeys treated by LV [19]. The inefficiency of the LM-PCR method can be caused by the burden of the multiple methodological steps. Our results also show that the genomic context may perturb the amplification of certain genomic regions around specific IS, and thus reduce the efficiency. This problem should not affect the DNA barcoding method which relies on the amplification of vector tags [19]. However, the reported barcoding technique does not provide the genomic position of the integrations. Thus, while the IS analysis by LM-PCR continues to be a method of choice to evaluate the safety of gene therapy in humans, technical improvements would be needed for better coverage and efficiency. More sensitive methods for IS retrieval relying on PCR amplification will probably suffer the same amplification or sequencing bias as current methods. Identification of vector insertion site from each LTR of the integrated provirus may be another improvement as the genomic context may be more favorable at one or the other end. We used this strategy to confirm the IS identity of clone KS40 on chromosome 2 using specific PCR and SANGER sequencing from the 5′LTR (not shown). The recently reported CReVIS-Seq method based on CRISPR/cas9 induced cleavage of genomic fragments containing LTRs, is able to detect both 3′ and 5′ viral DNA/genome junctions, also reduces PCR biases and provides possibilities for multiplexing targets other than LTRs for a more in-depth analysis of lentiviral IS [20].

Lentiviral standard cloned cell lines are therefore useful quality control materials to validate new methods. Such standards are clearly more pertinent materials than plasmids as they can control for all the steps applied to the sample of gene-modified cells, including DNA extraction as well as PCR steps. They also ensure a more reproducible and well-defined reference material than batches of gDNA made from bulk populations of transduced cells. In our laboratory, the new standards established from diploid HCT116 cells have replaced the previously reported HT1080 cell standards [9] which had drifted genetically [14]. We chose to establish a panel of three clones containing 1, 2, or 3 integrated proviruses since the values correspond to the generally-expected VCN values in clinical gene therapy trials. Additional cell lines with 1 to 5 VCN were obtained, partly characterized and cryopreserved. There is an international effort to standardize VCN measures and IS analysis. A panel of three lentiviral standards comprising a negative control without lentiviral integration, a single copy cell line and a third with five copies of lentiviral genome was established to be distributed through the World Health Organization standardization Committee and the National Institute for Biological Standards and Controls [12]. Others have reported a panel of four standards with 1, 2, 3, or 4 copies of lentiviral vector per cell [10]. Having additional standards like ours might be useful to the community. While standards established previously by us and by others express the GFP transgene [9, 10, 12] our new standards express the dNGFR transgene which can be detected by cytometry and serve for the positive selection of transgene-expressing cells using magnetic beads, should this be needed. The new standards could be used rather universally as quality controls because the integrated lentiviral cassette contains several features enabling them to control for vectors of second or third generation and match with the various primer sequences used for other published VCN and titration assays and targeting the HIV psi, gag, RRE, and LTR sequences [9, 10, 12]. A diverse panel of cellular standards may be valuable as there may not be an ideal universal standard. Indeed, as seen in the vector IS study the amplification of IS may depend on the genomic context. Thus, our standards could be compared and tested with others to become comparative reference materials for the international community. Reference material will become more and more necessary to calibrate and to validate VCN assays for gene therapy trials, or new technologies, in particular to compare results from different laboratories in multicentric trials and ultimately for the commercialization of products.

## Supplementary information

Supplemental materials
