# Supplementary material for: Lentiviral standards to determine the sensitivity of assays that quantify lentiviral vector copy numbers and genomic insertion sites in cells
Source: Gene Ther. 2022 Feb 22;29(9):536–43. doi: 10.1038/s41434-022-00315-8 (PMC9482878; doi:10.1038/s41434-022-00315-8)
Supplement: Supplementary file 1 — Supplemental materials [file 41434_2022_315_MOESM1_ESM.docx]

**SUPPLEMENTARY MATERIALS**

**Supplementary methods**

**Supplementary table S1**

**Chromosome spreads and counting**

Exponentially growing cells were treated with nocodazol (0.1µg/ml, sigma # M1404) during 3 hours then subjected to an hypotonic choc using 5ml of ice-cold KCl 0.56% for 6 min at room temperature, centrifuged (4 min, 800g), and the cell pellet was fixed by 5ml of methanol/acetic acid (3:1 v/v) added drop-by-drop under constant mild agitation. Chromosome spreads were obtained by depositing drops of cell suspension on a microscope slide in high humidity condition, air-drying the drops for 30 min and mounting the preparations using Fluoromount-G ®/DAPI (Southern Biotech, Birmingham, AL). Chromosome spreads were photographed using a 63x microscope immersion objective (Zeiss axioplan2, Oberkochen Germany). Around 25 well-separated chromosome spreads per slice with well-condensed chromosomes were analyzed manually using the imageJ image-processing program (V1.52).

**Supplementary Figure S1**

**
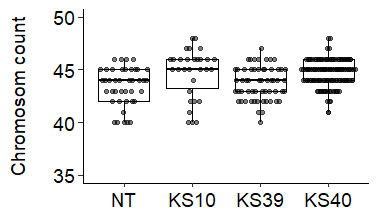
**

**Supplementary Figure S1 legend : Ploidy evaluation in selected clones.**

A chromosome count was performed on the 3 selected reference clones and un-transduced HCT116 cells using a microscopic manual method. For each cell population, more than 25 metaphases were analyzed.

**Supplementary Tables**

**Supplementary Table 1 : Sequences of primers and oligonucleotides used in the study**

| Oligo | Sequence 5’ –> 3’ |
| --- | --- |
| Alb.fw | GCTGTCATCTCTTGTGGGCTGT |
| Alb.rv | ACTCATGGGAGCTGCTGGTTC |
| Alb.pr | FAM-CGCACGGCAAGAGGCGAGG-QSY |
| Psi.fw | CAGGACTCGGCTTGCTGAAG |
| Psi.rv | TCCCCCGCTTAATACTGACG |
| Psi.pr | FAM-CGCACGGCAAGAGGCGAGG-QSY |
| PRO.fw | CACTCCCAACGAAGACAAGA |
| PRO.rv | TCTGGTTTCCCTTTCGCTTT |
| PRO.pr | TCTCTAGCAGTGGCGCCCGAACAGG |
| Linker+ | GTAATACGACTCACTATAGGGCTCCGCTTAAGGGACT |
| Linker- | [Phosp]-GTCCCTTAAGCGGAG-[AmC7] |
| VISA1.vector | AGTGCTTCAAGTAGTGTGTGCC |
| VISA1.linker | GTAATACGACTCACTATAGGGC |
| Blocking oligo | iBNA-meC/A/iBNA-G//iBNA-G/GACTTGAAA/iBNA-G//iBNA-meC/GAAA/iBNA-G//iBNA-G//iBNA-G/AAA/iBNA-meC/C/3AmMO |
| VISA2.TAGx.vector | TCGTCGGCAGCGTCAGATGTGTATAAGAGACAGGTCTGTTGTGTGACTCTGGTAAC |
| VISA2.vector | GTCTGTTGTGTGACTCTGGTAAC |
| VISA2.linker | GTCTCGTGGGCTCGGAGATGTGTATAAGAGACAGAGGGCTCCGCTTAAGGGAC |

**Table S1 legend :** BNA = bridged nucleic acid ; meC= methyl cytosine; AmMO amino modifier
